# Data retrieval and harmonisation for individual participant data meta-analysis in the context of a neglected tropical disease: challenges and lessons from the Infectious Diseases Data Observatory visceral leishmaniasis data platform

**DOI:** 10.1136/bmjph-2025-003986

**Published:** 2026-08-04

**Authors:** Prabin Dahal, Sauman Singh Phulgenda, Gemma Buck, Kasia Stepniewska, Philippe J Guerin

**Affiliations:** 1 Infectious Diseases Data Observatory (IDDO), Oxford, UK; 2 Centre for Tropical Medicine and Global Health, Nuffield Department of Medicine, University of Oxford, Oxford, UK; 3 Coalition for Equitable Research in Low-Resource Settings (CERCLE), Paris, France

**Keywords:** Systematic Review, Epidemiology, Epidemiologic Research Design

## Abstract

**Introduction:**

Individual participant data meta-analysis (IPD-MA) is the gold-standard approach for evidence synthesis. Poverty-related infectious diseases, a context in which standalone trials often have small sample sizes and consequently observe relatively few treatment failures, can benefit from this approach.

**Methods:**

In collaboration with the global research community, the Infectious Diseases Data Observatory (IDDO) has developed a data platform for visceral leishmaniasis (VL)—a neglected tropical disease (NTD), primarily to facilitate IPD-MA aimed at addressing questions of public health importance. Initially, a systematic review (SR) was undertaken to identify relevant trials since 1980 and study authors were invited to collaboratively develop the platform and participate in two IPD-MAs. IPD non-retrievability (data loss) was tracked and defined as unsuccessful attempts to contact study authors, or when the authors explicitly stated data loss, or declined participation.

**Results:**

The SR identified 147 VL trials (1983–2019), of which IPD was shared to the IDDO platform from 31 (21.1%). Reasons for data non-retrievability of 116 studies included: authors’ retirement (n=42, 36.2%), data were lost (n=30, 25.9%), authors’ non-response (n=22, 19.0%), authors were no longer contactable (n=17, 14.7%) and a lack of clarity over data ownership (n=5, 4.3%). The median sample size was 226 (IQR: 120–542, range: 30–1,143) for retrieved studies compared with 81 (IQR: 34–151, range: 7–3,126) for non-retrieved studies. No IPD were retrieved from trials published pre-2000 (0%, 0/63). Following solicitation, the median time to complete data harmonisation was 633 days (n=29 studies).

**Conclusions:**

Data solicitation and harmonisation steps remain underappreciated challenges in undertaking IPD-MA, particularly for NTDs where resources are severely limited. A critical mass of IPD from historical VL trials is no longer retrievable. It is imperative that further loss of such valuable data from ongoing and future trials is prevented.

**WHAT IS ALREADY KNOWN ON THIS TOPIC**

- Individual participant data meta-analysis (IPD-MA) is the gold-standard approach for evidence synthesis.
- Several challenges can be anticipated along various phases of planning and execution of IPD-MA, such as developing data sharing pipelines, harmonisation of studies into a common data format, maintaining data confidentiality and complying with privacy regulations—all of these require substantial planning and resources upfront, and can prolong the anticipated timelines for the completion of the IPD-MA.

**WHAT THIS STUDY ADDS**

- We detail our experience of undertaking IPD-MA in the context of visceral leishmaniasis (VL)—one of the poverty-related neglected tropical diseases, and share our unique experience of collaboratively developing the Infectious Diseases Data Observatory (IDDO) VL data platform with the ultimate aim of facilitating IPD-MAs.
- An initial systematic review identified 147 relevant studies (1983–2019) which were targeted for IPD-MA on drug efficacy and safety. IPD was shared to the IDDO VL data platform from a fifth of the targeted studies.
- Our study provides context-specific reasons for data non-retrievability. Main reasons for data non-availability included: study authors’ retirement, age of the publication, that is, publication prior to 2000, study authors stating data were no longer retrievable and non-response to the invitation to participate in the proposed IPD-MAs.

**HOW THIS STUDY MIGHT AFFECT RESEARCH, PRACTICE OR POLICY**

- A critical mass of data from historical VL trials has been lost and remains no longer retrievable to the research community.
- It is imperative ro prevent further loss of valuable data from ongoing and future trials. Open access and carefully governed repositories, such as the IDDO platform, built on the premise of purpose-driven data sharing and equitable partnership can serve as a scientific commons and a neutral platform.

## Introduction

 Individual participant data meta-analysis (IPD-MA) overcomes several shortcomings associated with meta-analysis of aggregated data.^1–4^ The latter uses data at a study (or treatment arm) level, most often extracted from a publication and relies on the completeness of reporting and is affected by heterogeneity in outcome measures adopted.^1^ For example, in the context of malaria, substantial heterogeneity in the definition of malnutrition and the reported effect measures precluded an aggregate data meta-analysis.^5^ An IPD-MA was subsequently undertaken on the same topic that investigated the role of acute malnutrition in antimalarial treatment outcomes.^6^ The availability of IPD allowed harmonisation of underlying studies to a common unit/scales of measurement, and adoption of a uniform definition of exposure and outcome across all the studies.

IPD-MA also enables investigators to address research questions that a standalone trial may not otherwise permit. For example, investigating the relationship between a particular subgroup and treatment outcome is typically subject to aggregation bias—the availability of IPD in such situations allows overcoming this limitation. This is particularly relevant for poverty-related infectious diseases such as malaria and neglected tropical diseases (NTDs). In these contexts, standalone efficacy trials have relatively small sample sizes,^7–9^ and subsequently the number of treatment failures observed in any single trial is also typically small.^10–12^ IPD-MA provides a unique opportunity to investigate the putative factors of therapeutic outcomes or investigate treatment effect in a subpopulation of interest, which would otherwise not be possible in a single trial or would necessitate undertaking a large clinical trial.

Undertaking IPD-MA, however, is a resource-intensive process and requires meticulous planning and considerations (online supplemental table S1). Several challenges can be anticipated along various steps involved, such as the details of the contacting authors or their emails may no longer be relevant, in which case further effort is required to establish a connection with the study authors/institutions. Even when a connection is established, IPD from the eligible studies may not be retrievable at all, or the study authors may decline participation. Once study investigators agree to participate, further data governance aspects such as confidentiality, delineating data ownership and developing an equitable data sharing agreement take considerable time and effort. These steps usually require careful upfront planning. Complex negotiations may be required to reassure the primary study authors, and differences in data protection regulations across countries and cultures should also be taken into consideration. And when data are finally shared for the purpose of the research, they may be in different file formats (languages, variable naming conventions) across the studies. Further checks are required to assess the completeness of the submitted data, corroboration against that reported in the publication and further involvement of the primary study authors is required for resolution of data queries. All of these steps can add significant delays to the anticipated timelines and add to the overall cost of undertaking such an endeavour.

Visceral leishmaniasis (VL) is one of the poverty-related NTDs targeted by the WHO for elimination by the year 2030. Availability of a consolidated and harmonised database of historical clinical trials can facilitate the generation of high-quality evidence on different aspects of drug efficacy, and allow identification of rare safety signals, which typically require a large volume of data. Together, these contributions remain important towards achieving the 2030 targets. In this manuscript, we share our experience of collaboratively developing the Infectious Diseases Data Observatory (IDDO) VL data platform as a common scientific resource with the ultimate aim of facilitating IPD-MAs to address questions of public health importance on drug safety and efficacy. In particular, we share the context-specific challenges in identifying and contacting data custodians, retrieving trial IPDs and some of the challenges faced during data standardisation.

## Materials and methods

### The Infectious Diseases Data Observatory

IDDO has collaboratively engaged with multiple NTDs research community over the past decade to map the landscape of clinical trials for several infectious diseases.^13^ These mapping exercises have assessed the feasibility of developing a data platform that can subsequently facilitate IPD-MA to address questions of public health importance, in particular regarding drug resistance, which remains a persistent problem across many infectious diseases. Therefore, IDDO’s primary focus has been to promote IPD-MA to investigate different aspects of drug dosing and safety, which can subsequently contribute towards maximising the therapeutic shelf-life of existing drugs. To achieve this, IDDO has actively engaged with the research community by forming a disease-specific scientific advisory committee (SAC). The SAC provides overall scientific steering and guides the identification of research questions to be addressed using IDDO’s data platform.

IDDO’s data platform is built on the premise of a transparent and equitable data ownership model. Under this model, the investigators who generated the primary study remain the data controller. They can engage, if they want, in subsequent projects when their study data are reused for addressing new research questions^14^; this governance framework is described in the literature and regularly updated on the IDDO’s website.^15–17^ This means the primary investigators who generated the data are engaged across multiple stages of the proposed IPD-MA, such as contributing to the protocol development, drafting the analysis plan, interpretation of the results and contributing to the scientific publication.^14^ Under IDDO’s umbrella, several scientific collaborations have informed the treatment guidelines on malaria over the past decade.^18–21^ For the VL theme, IDDO is currently collaborating with the research community to undertake IPD-MA from clinical trials aimed at drug safety and efficacy and developing a prognostic model for supporting risk-stratification of patients (further description presented on the webpage^22^), and an additional individual-level analysis in the context of Brazil using the national registry database.^23^

### VL and the need for a data platform

NTDs disproportionately affect the ‘bottom billion’ population of the world,^24^ primarily women and children and impoverished communities from the developing countries.^25^ Among parasitic diseases, VL inflicts the largest mortality burden after malaria.^24^ A key therapeutic outcome in assessing antileishmanial efficacy is ‘relapse’—which is the recurrence of the disease after achievement of initial cure. In VL, it is widely accepted that treatment alone doesn’t provide sterile cure and a vital contribution of cell-mediated immunity is expected.^26^ Therefore, patient factors are expected to play an important role in determining subsequent treatment outcomes. In clinical trials, approximately 3%–5% of the treated patients are expected to relapse, and there is observed geographical heterogeneity in drug response.^12^ For example, single-dose amphotericin B, which has demonstrated high efficacy in the Indian subcontinent (ISC), has been found to be less efficacious in the context of East Africa.^27,28^ VL trials are typically small,^7^ hence the possibility of investigating factors associated with treatment relapse or studying dose–response relationships in a single study remains impossible unless a large clinical trial is undertaken. Careful synthesis of IPD of existing studies provides opportunities to address these questions—this was the major scientific impetus for developing a global VL data platform.

### Development of the IDDO VL data platform: some key steps

The development of the IDDO VL data platform had three key steps: a scoping phase, an IPD-platform development stage and a research agenda development phase aimed at identifying key questions which the platform could address.

#### Scoping phase

As a first step towards the development of a data platform, a systematic review (SR) was undertaken in 2017 to identify prospective VL efficacy studies. The SR identified 145 trials with more than 25,000 patients enrolled; this informed the development of an IPD platform was feasible and such a platform was deemed to add value to the scientific community.^7^

#### Pilot phase of the platform development

Following the scoping phase, IDDO engaged with the research community by convening a SAC comprising of VL scientists and epidemiologists to guide the subsequent phases of the platform development.^29^ The VL SAC members initially contributed IPD from their trials in a pilot phase to support the development of a common data standard and identify areas that required consensus agreement (such as adopting outcome definitions). An IDDO implementation of the Clinical Data Interchange Standards Consortium (CDISC)-compliant format was chosen as the common standard for IPD harmonisation. To address the VL-specific nuances, custom domains were developed in collaboration with the scientific community and the CDISC experts. The tailored development required several iterations before the standard was finalised.

#### Development of a research agenda and identification of questions for IPD-MA

As the IPD platform was under development, concurrently a research agenda was developed in consultation with the VL SAC, global research community and key stakeholders. The primary focus of the research agenda was to solicit priority research questions from the global research community that could be addressed using the IPD platform. An account detailing the iterative development of the research agenda and a summary of the inventory of the research questions identified is described elsewhere.^30^ From the research agenda, the VL SAC endorsed two questions to be addressed using an IPD-MA: (1) the evolution of haemoglobin profile during the treatment and convalescence phase, and (2) identification of factors associated with treatment failure (relapse).^22^ The former IPD-MA was important as there is a marked heterogeneity in the haematological parameters adopted across the clinical trials for patient enrolment.^31^ Such heterogeneity precluded possibility of an aggregate meta-analysis. Furthermore, haemoglobin improvement is generally observed within 4–6 weeks of treatment, but the impact of parasite load, patient’s age and drug regimen on recovery trajectory remains poorly understood. The SAC deemed addressing the questions on haemoglobin recovery using the IPD-MA was feasible through the data platform. Similarly, the second IPD-MA focused on treatment relapse, which is the subsequent recurrence of the disease (typically assessed with a 6-month follow-up) after achievement of initial cure (typically assessed at around 1 month). Relapse remains an important public health priority as they constitute a parasitic reservoir and is associated with increased infectivity.^32^ Clinical trials have shown a geographical variation in relapse patterns; therefore, the VL SAC recommended a region-specific IPD-MA to identify predictors associated with relapse and other treatment outcomes.

The next step was then to invite the authors of the studies identified from the SR to collaboratively participate in the two IPD-MAs. An updated SR was undertaken in 2021, and there were 147 clinical trials eligible for the purpose of the two proposed IPD-MAs (PROSPERO ID: CRD42021284622).^33^ The underlying statistical analysis protocols for these two IPD-MAs are publicly available and present further details.^34,35^

### Soliciting IPD for the purpose of the two proposed IPD meta-analyses

Between June 2018 and March 2021, IPD from 38 studies were submitted (including some unpublished) to the IDDO VL data platform during the pilot phase by the members of the SAC. Of these 38 studies, 27 could be successfully linked to one of the 147 eligible publications for the proposed IPD-MAs. Data contributors of these 27 studies were formally approached requesting their participation in the two IPD-MAs. For the remaining 120 studies, the trial investigators (first and last authors/corresponding authors initially) were contacted requesting their participation in the IPD-MA along with: (1) the proposed research questions outlining the rationale and importance, (2) a detailed data sharing agreement statement and (3) a list of the minimum variables requested. If there was no response from the first/last/corresponding authors of the study, then attempts were made to reach out to other study authors, if contact information (email) could be identified. An initial request was sent in April 2021 to the investigators, with a further two reminder emails sent approximately every 2 weeks. If the emails remained undelivered or no response was received after two follow-ups, then additional efforts were taken to reach out to these authors through the IDDO’s scientific networks before studies were finally considered to be non-retrievable.

### Assessment of non-retrievability

IPD non-retrievability (data loss) was defined as unsuccessful attempts to contact study authors, or when authors explicitly stated data loss or declined participation in the IPD-MA for any stated or unstated reasons.

### Time taken for retrieval and harmonisation of studies

The time between the initial email sent to the study authors requesting participation in the development of the VL data platform and the date when the study was eventually submitted to the IDDO data platform was recorded. In addition, we also kept detailed records of the time taken between the submission of the studies to the data platform and the completion of the harmonisation of the study to the CDISC-compliant standard.

### Patient and public involvement

No patients or members of the public were involved in this research.

## Results

**Figure 1:**
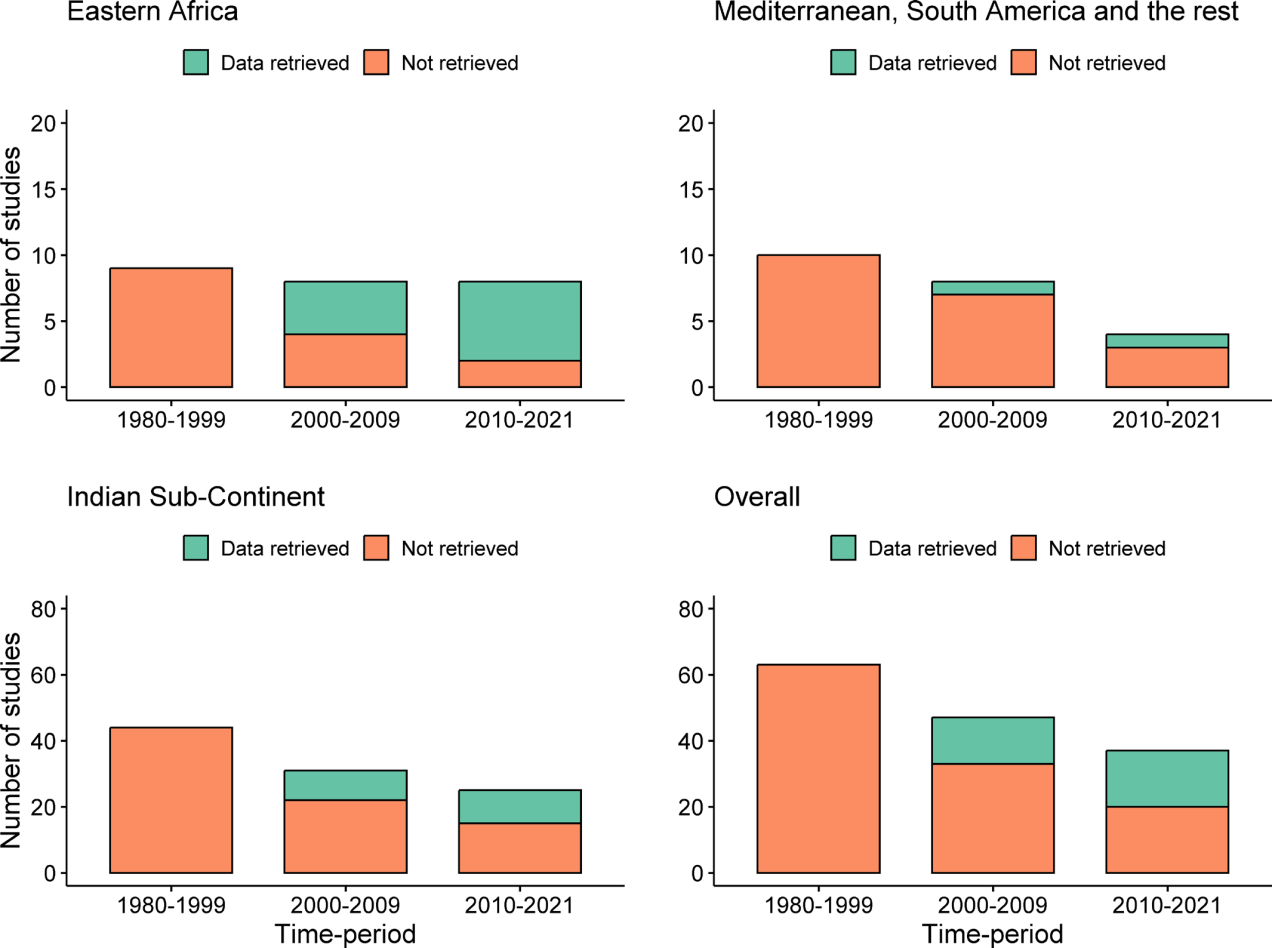
Data retrieval status of the studies included in the systematic review (n=147 studies).

Of the 147 targeted studies, IPD from 31 (21.1%) studies from nine different research institutions were shared with the IDDO VL data platform (figure 1). Of the 31 studies, 27 were already part of the IDDO VL data platform when the two IPD-MAs were formally initiated in April 2021. These studies were submitted by the VL SAC members during the pilot phase of the platform development.

### Characteristics of studies by retrieval status

**Figure 2:**
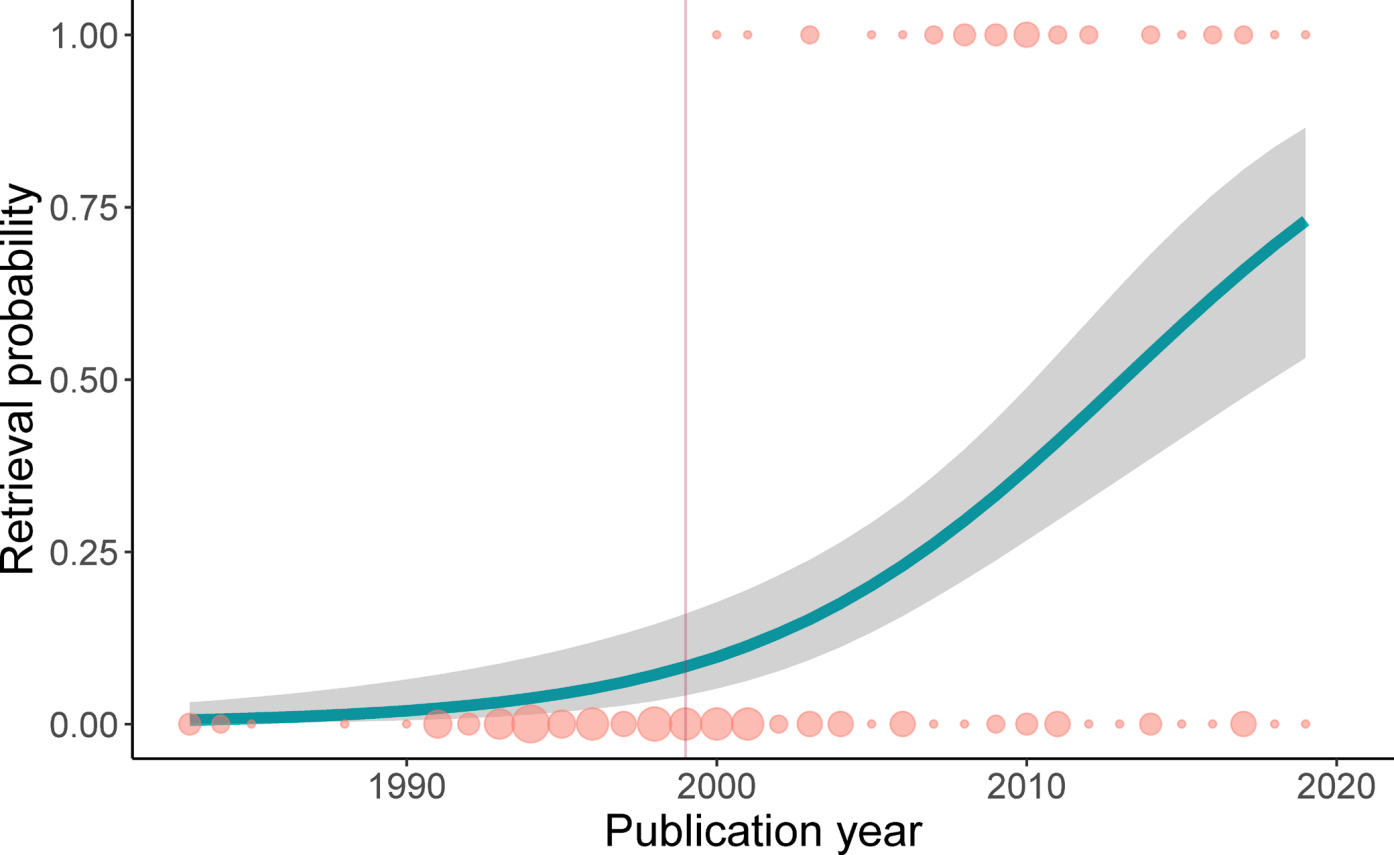
Probability of data retrieval over time (n=147 studies). Each circle represents the number of studies published (the size is proportional to the number of studies published in the given year). The vertical line is at year 1999, before which no data were retrievable for the proposed individual patient data meta-analysis. The blue line presents the estimate of probability of data retrieval obtained using a logistic regression (with publication year as the only predictor). The shaded region encloses the 95% confidence interval computed using Wald’s approach.

The median study sample size was 226 (IQR: 120–542, range: 30–1143) for the 31 retrievable studies, and this was 81 (IQR: 34–151, range: 7–3126) in 116 studies that couldn’t be retrieved (table 1). There were 63 studies published between 1980 and 1999, and none (0%) of them were retrieved; this was consistent across the regions (figures 1 and 2, table 1). There were 47 studies published between 2000 and 2009, of which 14 (29.8%) were retrieved. There were 37 studies published between 2010 and 2021, of which 17 (45.9%) were retrieved. None (0%) of the three studies from Central Asia could be retrieved; 10 (41.7%) of the 24 studies from East Africa were retrievable. Proportionately, the majority of the studies conducted after 2010 from the East African region and a fraction of the studies from the Indian subcontinent during the same time period were retrievable.

**Table 1:** Characteristics of the studies by retrieval status (n=147 studies)

	All studies (n=147)	Not retrieved (n=116 studies)	Retrieved (n=31 studies)
Median (IQR) sample size Range	101 (IQR 44–245) Range: 7–3126	81 (IQR 34–151) Range: 7–3126	226 (IQR 120–542) Range: 30–1143
Publication period			
1980–1999	63	63 (100%)	0 (0%)
2000–2009	47	33 (70.2%)	14 (29.8%)
2010–2021	37	20 (54.1%)	17 (45.9%)
Geographical region			
Central Asia	3	3 (100%)	0 (0%)
Eastern Africa	24	14 (58.3%)	10 (41.7%)
Indian subcontinent	100	81 (81.0%)	19 (19.0%)
Mediterranean region	8	7 (87.5%)	1 (12.5%)
Multiregional studies	3	3 (100%)	0 (0%)
South America	7	6 (85.7%)	1 (14.3%)
Unclear region	2	2 (100%)	0 (0%)
Randomisation status			
Randomised	62	47 (75.8%)	15 (24.2%)
Non-randomised (multiple arms)	25	18 (72.0%)	7 (28.0%)
Non-randomised (single group)	49	40 (81.6%)	9 (18.4%)
Notspecified	11	11 (100%)	0 (0%)
Patient age range			
Adolescent children and adults (≥15 years)	8	7 (87.5%)	1 (12.5%)
All ages	121	95 (78.5%)	26 (21.5%)
Less than 15 years	13	11 (84.6%)	2 (15.4%)
Unclear	5	3 (60%)	2 (40%)

Row percentages presented in parentheses.

### Reasons for data non-retrieval

Of the 116 studies that couldn’t be retrieved, the reasons for data not being shared included: authors of 42 studies (36.2%) had retired, data from 30 (25.9%) studies were described as old and lost and couldn’t be retrieved any longer, authors from 22 (19.0%) studies didn’t respond to any of the email requests, the contact emails of authors were not up-to-date and alternative emails could not be traced for 17 (14.7%), authors from 5 (4.3%) studies were willing to collaborate but were unable to obtain institutional clearance for the collaborative engagement as there was no clarity regarding ownership of data as intellectual property (this included one study where the authors agreed to participate but didn’t lead to the data submission) (online supplemental figure S1).

### Time taken for completion of curation

Once the IPD were submitted to the IDDO platform, the median time taken to complete the data standardisation, mesaured from the start of the curation to completion, for the studies eligible for the two IPD-MAs was 633 days (IQR: 382–804, range: 60–1,369) (online supplemental figure S2). These were due to the limited funding available for explicitly undertaking the IPD-MA initially, ongoing development and IDDO implementation of the CDISC-compliant data format during the early stages, and also due to communication delays as multiple email exchanges were required with the primary study investigators for resolution of queries and clarifications for completing data harmonisation. We kept detailed query records for 22 of these studies; a total of 53 data-related queries were made over 77 email exchanges with the study investigators to complete the standardisation of the IPD.

## Discussion

The research and development of novel drugs, diagnostics and vaccines for NTDs are marked by a chronic lack of funding.^36–39^ Trials with small sample sizes and a limited set of interventional trials are common features of the NTDs landscape. In the context of NTDs such as VL, an IPD-MA offers opportunities to address scientific questions that would otherwise not be possible in a single study or through an aggregate data meta-analysis, such as the development of allometric dosing for miltefosine, which was based on a pooled analysis.^40^ We attempted to develop a data platform by targeting VL studies published since 1980 with the aim of facilitating IPD-MA. The platform development had some key phases such as systematic scoping of the literature,^7^ convening a committee (SAC) to provide scientific steering and guide the iterative development of a research agenda^30^ and identifying questions which could be potentially addressed through an IPD-MA.^29^ Despite these extensive engagements, IPD retrieval was possible in only one-fifth of the targeted studies. The non-retrievability of a critical mass of historical data is a major loss to the research and patient community and severely limits the possibility of generating evidence regarding drug safety and efficacy on targeted subgroups (such as infants, the elderly or those with comorbidities such as TB). The low success observed echoes the findings of Nevitt *et al,* who found nearly 40% of the IPD-MA in infectious diseases retrieved <80% of the targeted studies (online supplemental table S2).^4^

In particular, of the 147 studies targeted, IPD from none of the studies published prior to 2000 could be retrieved. This adds to previous reports of diminishing retrieval probability with the age of publication^4,41^ and points towards the need to make a concerted effort to avoid loss of such valuable data in the future. Relatively better success in retrieval of data from recent studies could reflect the shifting view towards open science. This is highlighted by several important initiatives over the past decades, such as the Declaration on Research Assessment^42^ and the Paris Call on Research Assessment.^43^ Funding agencies and scientific journals now increasingly expect that investigators either make the data available publicly through a trusted repository or through a controlled data access system to promote open science and replicable research. Other major reasons influencing the non-retrievability of data were the retirement of the primary/contacting authors, authors’ non-response or authors were no longer contactable. For a number of historical studies, data were available in paper format, and the economic cost of digitisation remained beyond the scope of available resources. Furthermore, a lack of understanding and confusion over the ownership of data were also a reason leading to institutional barriers to data sharing, even when either the study authors or the sponsors were willing to contribute. Overall, these findings reflect the reasons typically reported in the literature.^4,44,45^

A majority of the retrieved datasets (27/31) shared to the IDDO platform came from researchers who were already part of the VL SAC and believed in the importance of establishing a global repository.^29^ A key aspect of such an undertaking was the adoption of an equitable approach across all aspects of the platform development. First, the SAC was constituted of global VL experts primarily based and working in the endemic countries. They provided scientific steering and led the development of the agenda for future research to be undertaken through the platform. Second, under IDDO’s governance framework, the primary data generators remain the data controller, and they can decide to either retain the right of future reuse or delegate this responsibility to an independent data access committee, currently chaired by the WHO/TDR, the Special Programme for Research and Training in Tropical Diseases.^46^ Finally, the primary investigators are also part of the subsequent research projects that use their data and can remain actively engaged in analysis, interpretation and drafting of the scientific manuscript. Overall, our experience highlights the importance of trust building and the need for stakeholder engagement for undertaking IPD-MA. This remains particularly important in the context of NTDs, as there are additional barriers compared with a well-resourced context. Some of the challenges include setting up international data transfer agreements, developing a transparent and equitable partnership, and soliciting the underlying data dictionary and protocol in addition to IPD. All of these add to the time and resources required to successfully retrieve and harmonise datasets. These challenges may be less of an issue in the context of a well-resourced setting, where funders have been promoting open access to data for a number of years and investigators have had the opportunity to factor in the associated costs. In addition, global health research has long suffered from chronic underfunding, and this has been exacerbated by the recent cuts, which require all the stakeholders to demonstrate solidarity and collaboration.^47^

After submission of the data to the platform, the major challenge encountered was the time taken to standardise (curate) the studies. The median time required to standardise was 633 days for studies that were eligible for inclusion in the two proposed IPD-MAs. Several factors contributed to the long time required for curation. Initially, limited resource were available for the curation support to meet the demanding structure of the CDISC-compliant format. Custom domains that remained CDISC-compliant had to be developed, and custom variables had to be added when no suitable options were available. This involved several rounds of discussion and review before the standards adopted for harmonisation were finalised. There was also a considerable delay in responses between data managers and the study contributors, along with multiple email exchanges required for query resolutions. In addition, primary data contributors having to retrospectively respond to the queries on the studies that were completed a considerable time ago could have played a role. This finding is similar to that reported by Ventresca *et al* study with 6.5 years required for data collation and analysis.^48^ Our observation, together with the reports from the literature,^2,48,49^ reflects that data preparation, curation, and harmonisation are underappreciated aspects of IPD-MA. Time and resources required for these steps should be appropriately factored in during the planning and budgeting stages.

There are some specific limitations to our study that should be considered. First, non-retrievability should be understood specifically in the context of data not shared to the IDDO data platform and for the purpose of the two proposed IPD-MAs. A vast majority of the studies that could not be retrieved were from scientists who had either retired or passed away, and data from these studies can be considered as lost. However, there was no engagement in some cases despite repeated requests, and some of the study investigators still had the data available either in paper format or lacked clarity regarding data ownership—the data from these studies may not be strictly lost *per se*. Second, the long time taken for standardisation of the studies reflects a combination of lack of funds available and the initial time investment required to collaboratively develop a custom CDISC-compliant format. Future harmonisation efforts are expected to be much faster given the maturity of infrastructure currently in place. Third, given the nuances and challenges in undertaking IPD-MA in the context of NTDs, the generalisability of our observations to a non-NTD context warrants caution. However, literature is now emerging regarding the demanding resources required for data solicitation and harmonisation steps^2,48^ and regarding the trial characteristics of the non-retrievable studies.^50^

Recent years have seen a proliferation of several public databases to host clinical trials data. However, IDDO’s approach presents a unique case study as the platform was developed collaboratively by engaging the disease research community and key stakeholders, by developing a transparent, equitable governance framework outlining the data ownership and reuse model. In particular, the platform’s approach of purpose-driven data sharing^17^ and the subsequent standardisation of data to a disease-agnostic CDISC-compliant format not only aligns well with the FAIR (Findable, Accessible, Interoperable and Reusable) data principles but also saves time and resources required to undertake future IPD-MA. Such resources spent upfront remain important to maximise the scientific reuse of the data from historical studies and are crucial in the context of NTDs due to financial and geopolitical stability constraints, especially as the global health community is working towards the 2030 targets. The unique challenges and experiences encountered with the VL platform development described in this manuscript may serve as an exemplar for researchers working in similar disciplines and may help to avoid future data loss (box 1).

Box 1 **Suggestions for preventing data loss in the future**

**Individual investigators**

- Register the trial in a timely manner, and make the trial protocol and statistical analysis protocol publicly available.
- Once the trial is complete, store data within the institutional server (or within an appropriate repository while maintaining patient privacy).
- Create institutional memory: store associated protocol and documentation to prevent future loss. This will ensure that even when an investigator leaves the institution or retires, sufficient documentation is retained and there is clear information regarding the study to support future reuse.

**Funders**

- Create a dedicated funding line to support data infrastructure (eg, support server costs, specialised data management tools and software).
- Create data management roles and training opportunities.

**Journals**

- Strictly enforce data availability statements with clear instructions to avoid ambiguity. This can include enforcing a clear description of contact details for data requests, together with a description of institutional-specific policies of which the data requesters should be aware of.
- Make sharing of data dictionary mandatory when the investigators deposit their data to a secure repository/server. Ensure that all the data and variables used for the journal are clearly described in the associated data dictionary.
- Make sharing of analysis code used for data management along with statistical analysis scripts mandatory (while preserving privacy)—these can be shared on institutional GitHub or other appropriate locations.

**All**

- Practice responsible, ethical and equitable data sharing.
- Avoid data submission simply as a checklist exercise to meet funders’ or journals’ requirements. Share adequate documentation regarding data-generating processes, data systems and codebooks to promote future reuse.
- Avoid submission of data to portals that simply act as ‘data dumpsters’.^51^

## Conclusions

In the context of VL, a large proportion of critical data generated over the past decades is no longer retrievable. This is a major loss to the scientific and patient communities. Data platforms like IDDO can not only serve as a secure repository but also as a neutral platform to promote cross-regional collaboration. This remains vital in the face of current adversities such as chronic underfunding, data scattered across myriad platforms without adequate documentation and standardisation, geopolitical instability and drug resistance that continues to threaten the research and development across many NTDs.

## Supplementary material

10.1136/bmjph-2025-003986 online supplemental file 1

## Data Availability

All data relevant to the study are included in the article or uploaded as supplementary information.
