# Supplementary material for: Data retrieval and harmonisation for individual participant data meta-analysis in the context of a neglected tropical disease: challenges and lessons from the Infectious Diseases Data Observatory visceral leishmaniasis data platform
Source: BMJ Public Health. 2026 Aug 4;4(3):e003986. doi: 10.1136/bmjph-2025-003986 (PMC13448620; doi:10.1136/bmjph-2025-003986)
Supplement: Supplementary file 1 [file bmjph-4-3-s001.docx]

**Supplemental File 1:** Data retrieval and harmonisation for individual participant data meta-analysis in the context of a neglected tropical disease: Challenges and lessons from the Infectious Diseases Data Observatory Visceral Leishmaniasis data platform

**Supplemental file listing**

**Supplemental Table S1:** Key steps outlining Infectious Diseases Data Observatory (IDDO)’s collaborative study group model.

**Supplemental Table S2:** IPD retrieval rates and reasons for IPD non-availability for the IDDO VL data platform presented along with the findings of Nevitt *et al.*, BMJ 2017;357: j1390.

**Supplemental Figure S1:** Reasons for data non-retrievability (n=147 studies)

**Legend:** Other (n=5) includes 1 study in which the study authors responded with agreement to participate but didn’t lead to data submission, and 4 studies where the authors had to seek permission from their respective institutions and had no subsequent follow-up.

**Supplemental Figure S2:** Time taken to standardise data from the 31 studies with IPD available

**Legend:** Study 18 and Study 23 are the same but were split into two for curation purposes and the data is presently separately. Horizontal dotted lines with arrow indicate the two studies that were not included in the IPD-MA and the curation was de-prioritised. The vertical line indicates the time when requests were sent to all the investigators of the 147 studies that were eligible for inclusion in the IPD meta-analysis.

**Supplemental Table S1: Key steps outlining Infectious Diseases Data Observatory (IDDO)’s collaborative study group model**

**Key steps outlining IDDO’s collaborative study group model**

- Framing of the research question (PICOT)
- Systematic identification of eligible studies
- Identifying and contacting the legal institutions, which is the data custodian of the data to request access e.g. principal investigator(s), research institutions, or the pharmaceutical industry
- Putting in place a contractual agreement to share the data
- Developing an *a priori* data management and statistical plan (DMSAP) in collaboration with the study investigators
- Harmonisation of data from all the shared studies to a common standard
- Analysis of the standardised data using appropriate model as described in the DMSAP
- Interpretation of the results and findings from the analysis in collaboration with the study investigators
- Finally, dissemination of the results

IDDO = Infectious Diseases Data Observatory: PICOT= Population, Intervention, Comparator, Outcome, Time

**Supplemental Table S2: IPD retrieval rates and reasons for IPD non-availability for the IDDO VL data platform presented along with the findings of Nevitt *et al.*, BMJ 2017;357: j1390.**

|  | Nevitt *et al.*  BMJ 2017;357: j1390 | This study  (IDDO VL platform and VL IPD-MA) |
| --- | --- | --- |
| **IPD-MA in infection and Infectious Diseases ^a^** |  |  |
| Denominator | 31 IPD-MA | 147 trials |
| Proportion of studies retrieved for IPD-MA | 100% retrieved (n=6, 19%)  ≥80% retrieved (n=9, 29%)  <80% retrieved (n=12, 39%)  Unknown (n=10, 32%) | 21.1%  (31/147) |
| **Reasons for unavailability of IPD ^a^** |  |  |
| *Denominator* | 571 IPD-MA without 100% of IPD retrieved | 116 non-retrieved  trials |
| Data not available | 341 (60%) | - |
| Investigators could not be contacted | 104 (18%) | 17 (11.6%), |
| Investigators declined to share data | 74 (13%) | - |
| Data lost or destroyed | 65 (11%) | 30 (20.4%) |
| Data could not be extracted | 55 (10%) | - |
| Trial was still ongoing | 42 (7%) | - |
| Data quality issues | 29 (5%) | - |
| Failed to provide data in time for IPD meta-analyses | 26 (5%) | - |
| Data not requested | 24 (4%) | - |
| Ethical or ownership restrictions | 15 (3%) | 5 (3.4%) |
| Reason unclear | 11 (2%) | - |
| Authors did not respond | - | 22 (15.0%) |
| Authors had retired | - | 42 (28.6%) |

IPD-MA = Individual participant data meta-analysis

**^a^** Note the unit of analysis is different for the Nevitt *et al.* and the IDDO VL IPD-MA. The former has IPD-MA as the unit of analysis and the VL IPD-MA as clinical trials as the unit of analysis.

**Supplemental Figure S1:** Reasons for data non-retrievability (n=147 studies)

Legend: Other (n=5) includes 1 study in which the study authors responded with agreement to participate but didn’t lead to data submission, and 4 studies where the authors had to seek permission from their respective institutions and had no subsequent follow-up.

**Supplemental Figure S2:** Time taken to standardise data from the 31 studies with IPD available

Legend: Study 18 and Study 23 are the same but were split into two for curation purposes and the data is presently separately. Horizontal dotted lines with arrow indicate the two studies that were not included in the IPD-MA and the curation was de-prioritised. The vertical line indicates the time when requests were sent to all the investigators of the 147 studies that were eligible for inclusion in the IPD meta-analysis.
